# Cholera

**Published:** 1848-10

**Authors:** 


### CHOLERA.

"In the whole of Russia," says the London Medical Times, "since the first appearance of the cholera, on the 28th of October, 1846, to the 5th of July, 1848, 290,318 persons were seized with the epidemic, and 116,658 died." According to late accounts, the disease was subsiding in Russia, but was spreading in Egypt, and some parts of Prussia. At Berlin it was quite rife. The Prussian government has established Cholera Hospitals in all the towns and large villages, and taken every care to lessen the horrors of the approaching pestilence.

"The French government has appointed a medical commission, composed of MM. Gueneau de Mussy, Chomel, Andral, Husson, Bouillaud, Bally, Gerardin, Cornac, and Gaultier de Claubry, to apply themselves to the discovery of means to prevent and to mitigate the effects of Asiatic Cholera."

